# Neuroinflammation and Neurodegenerative Diseases: How Much Do We Still Not Know?

**DOI:** 10.3390/brainsci14010019

**Published:** 2023-12-23

**Authors:** Carmela Rita Balistreri, Roberto Monastero

**Affiliations:** 1 Cellular and Molecular Laboratory, Department of Biomedicine, Neuroscience and Advanced Diagnostics (Bi.N.D.), University of Palermo, 90134 Palermo, Italy; 2 Unit of Neurology & Neuro-Physiopathology, Department of Biomedicine, Neuroscience, and Advanced Diagnostics (Bi.N.D), University of Palermo, Via La Loggia 1, 90129 Palermo, Italy; roberto.monastero@unipa.it

**Keywords:** neuroinflammation, neurodegenerative diseases, emerging mechanisms

## Abstract

The term “neuroinflammation” defines the typical inflammatory response of the brain closely related to the onset of many neurodegenerative diseases (NDs). Neuroinflammation is well known, but its mechanisms and pathways are not entirely comprehended. Some progresses have been achieved through many efforts and research. Consequently, new cellular and molecular mechanisms, diverse and conventional, are emerging. In listing some of those that will be the subject of our description and discussion, essential are the important roles of peripheral and infiltrated monocytes and clonotypic cells, alterations in the gut–brain axis, dysregulation of the apelinergic system, alterations in the endothelial glycocalyx of the endothelial component of neuronal vascular units, variations in expression of some genes and levels of the encoding molecules by the action of microRNAs (miRNAs), or other epigenetic factors and distinctive transcriptional factors, as well as the role of autophagy, ferroptosis, sex differences, and modifications in the circadian cycle. Such mechanisms can add significantly to understanding the complex etiological puzzle of neuroinflammation and ND. In addition, they could represent biomarkers and targets of ND, which is increasing in the elderly.

## 1. Introduction

The term “neuroinflammation” indicates representative pathological conditions induced in the brain by several (local and systemic) triggering factors (e.g., infections, trauma, ischemia, toxins, alterations in the microbiota–brain axis, etc.) and driving factors (e.g., genetic, vascular, and brain factors: for example, alterations in the expression of neurotrophins and components of the endothelial glycocalyx and/or endothelium) [1,2]. Neuroinflammation is evoked by typical immune cells residing in the brain and well known to have a key role in central nervous system (CNS) homeostasis and the development of neurodegenerative diseases (NDs), constituting their typical hallmark [3,4,5]. However, recent evidence suggests that both peripheral and infiltrating monocytes, as well as cells of clonotypic immunity, constitute other crucial actors of neuroinflammation [5,6]. Nevertheless, such evidence needs to be supported by larger studies, even if the growing data obtained in recent years appear promising and demonstrate the crucial contribution of this immune component in brain health and disease [3,4,5,6].

Other unconventional mechanisms related to the onset of neuroinflammation have recently emerged in the literature. Among these includes the altered relationship of the gut microbiota with the CNS, known as gut–brain microbiota (MGB) axis [7,8]. Of note also is autophagy, although the related mechanisms remain indefinable, and further investigation is necessary [9]. Interesting also is the contribution of ferroptosis, a novel cell death form caused by iron-dependent lipid peroxidation [10,11,12] and associated with the pathogenesis of many diseases, such as ND. Such has led some to suppose that variations in the iron metabolism’s homeostasis, the consequent induction of oxidative stress, and the related inflammation in the CNS is involved in the onset of ferroptosis and neuronal health [10,11,12]. In such a process, the apelinergic system, mediated by ELA/APJ signaling, has also been recently documented to participate in this regulation [13]. Another nonclassical mechanism related to onset of neuroinflammation appears to be the modified expression of neurotrophins, such as BDNF [14].

Furthermore, it is emerging that neuroinflammation is also the result of the modulation in expression of genes encoding immune and injury’s molecules. miRNAs [15] and epigenetic factors [16], including A-to-I RNA editing, M6A RNA methylation, and alternative splicing [15,16,17], have recently been revealed to have a fundamental role. Finally, circadian rhythm disorders have recently also been discovered to impact both the onset and development of neuroinflammation through the activation of glial cells and peripheral immune responses [18].

Insights have been achieved in identifying the mechanisms related to the complex neuroinflammation, although the complex puzzle is not complete and further studies are needed. Here, we describe and discuss the above mechanisms and others by reporting current clinical and experimental evidence.

## 2. Recent Evidence on Peripheral and Infiltrating Monocytes and Clonotypic Immune Cells in Neuroinflammation and Their Sex- and Gender-Mediated Modulation

**Figure 1:**
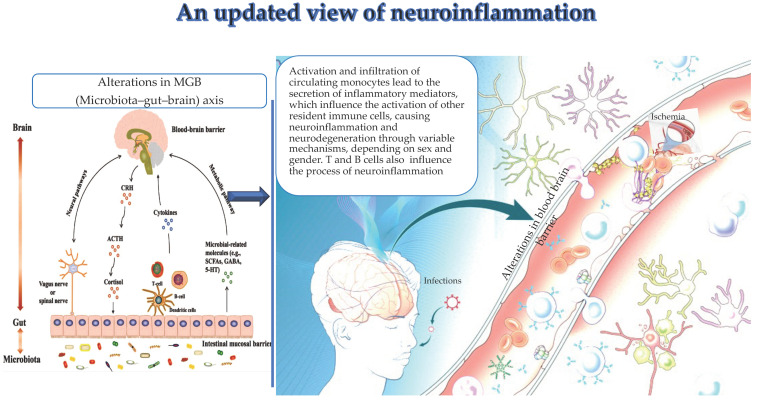
(by Biorender software version 04): Important drivers, i.e., alterations in the MGB axis, infections, and ischemia contribute to the activation and infiltration of circulating monocytes in the brain and their specific secretion of inflammation mediators. These latter influence the activation of other resident immune cells, thus leading to neuroinflammation and neurodegeneration. Similarly, clonotype cells influence neuroinflammation.

Monocytes and clonotypic immune cells represent key actors of neuroinflammation, as recently underlined (see Figure 1) [18,19,20,21]. Such cells physiologically protect by pathogens, and particularly confer resistance against neurotropic viruses [18,19,20,21]. In addition, they contribute to CNS physiological functions and structure, by controlling the development, and improving cognitive function. In ND conditions, immune cells, i.e., monocytes and clonotypic cells, are deregulated. The deregulation commonly impacts both the levels and functions of clonotypic cells and monocytes, with the consequent evocation of abnormal immune responses [4]. Accordingly, monocytes are frequently altered, both peripherally and centrally, in quantity and quality with altered profiles and phenotypes. Data from recent human and animal studies report that monocytes, different populations of lymphocytes, and their mediators can evoke both self-protective and injurious mechanisms in ND, perturbing both their progression and risk of neuronal death. This displays a close interplay of peripheral immune cells with those residents in the CNS, which significantly influences the evolution of ND and consequent survival in ND cases. Accordingly, changes in number or functional quality of peripheral macrophages can modulate inflammation at the periphery along nerves and in the CNS [18,19,20,21]. Extracellular vehicles (EVs) from misfolded proteins and mediators of inflammation released by the cells appear to have a fundamental function in the inflammatory amplification [6], as recently reported by the literature.

### 2.1. Monocytes in Neuroinflammation

The participation of monocytes in the evocation of neuroinflammation has recently emerged from both clinical studies and experimental observations [21,22,23,24,25]. Accordingly, the presence of infiltrated peripheral immune cells in mouse models with degenerative conditions, including Parkinson’s disease (PD), has been detected [21,22,23,24,25,26]. Infiltration of peripheral blood monocytes into the brain has also been observed in cerebrovascular diseases [27], as well as in patients with multiple sclerosis, where monocytes have been found to secrete some anti-neurodegenerative mediators [28]. In addition, in vitro studies have reported that high levels of chemokine CXCL12 from monocytes induce endothelial cell (EC) activation, thereby facilitating lymphocyte transmigration and validating the critical action of monocytes in the infiltration of immune cells in the brain [29]. Another study has established that brain immune infiltration originates from systemic inflammation [30]. Furthermore, circulating Ly-6C+ myeloid precursors have been observed to migrate into the CNS and have a pathogenic role in individuals affected by autoimmune demyelinating disease [31]. Moreover, monocytes have been demonstrated to phagocytose surplus brain proteins [32], such as amyloid-β peptide [33].

Other animal models, such as the murine stroke model, have evidenced a neuroprotective function of monocytes [34]. Precisely, they provide bioactive substances to brain cells [35,36]. In contrast, other investigations have reported the toxic action towards neuronal cells of monocytes by releasing saturated fatty acids, which can cause diverse pathologies, such as autoimmune disorders [21,22,23,24,25]. Thus, monocytes, through different mechanisms, actively contribute to the development of neuroinflammation, although some require further investigation.

### 2.2. Clonotypic Immune Cells in Neuroinflammation and ND

The role of clonotypic cells in neuroinflammation has only been theorized in previous research. However, recent evidence has largely established the key role of both T and B cell subsets and demonstrated their infiltration into the CNS by determining different effects depending on different subsets [37,38]. Therefore, regulatory T cells (Tregs) and Th2 cells have a neuroprotective effect. In contrast, Th1, Th17, cytotoxic T cells, natural killer (NK) cells mediate an accelerate progression of neuroinflammation, which can result in an exacerbated/accelerated neurodegeneration and an increased mortality risk [39,40,41,42]. Furthermore, they show different systemic levels. Precisely, circulating Th17 cells have shown higher levels in subjects with mild cognitive impairment (MCI) in cognitively normal subjects or those with non-Alzheimer’s MCI [42]. In contrast, circulating Th1 cells have been demonstrated to have higher levels in subjects with AD [43]. Th2 cells and Th2-associated molecules have been observed to have lower levels in AD subjects [43]. Other studies have reported higher levels of circulating Th17 populations in individuals with AD [44,45]. Treg cells have also been discovered to have lower levels in patients with AD [46]. They are anti-inflammatory, with opposite effects to Th17 cells. Fu et al. have described low levels of Treg cells in AD subjects [47]. Contrarily, another investigation has observed no difference in Treg levels between MCI, AD, and healthy subjects, although Treg levels are associated positively with total tau and pTau181 in AD subjects [48].

Different T subsets with diverse functions have been detected in individuals affected by amyotrophic lateral sclerosis (ALS), characterized by the gradual degeneration of upper and lower motor neurons. T cells from superoxide dismutase (SOD)1-mutant mice have been observed to enhance and evoke survival of motoneuron cells (MNs) through a defensive neuroinflammatory response, likely mediated by interleukin 4 (IL-4). In contrast, motor impairment is accompanied by a decline in the functions of Treg cells, which inhibit microglia activation in SOD1-mutant mice [49]. Consequently, functions of neuroprotection mediated by the immune cells may happen in the early stage of the disease, although other studies are needed to confirm this. Moreover, disease progression is linked to numerous changes in the immune system, including the acquisition of an inflammatory phenotype of microglia cells [50], thymic involution [51], augmented levels of proinflammatory cytokines [52], and CNS leukocyte infiltration [53].

There is inadequate evidence and inconsistent data in the literature on the function of B lymphocytes, plasma cells, and antibodies in neuroinflammation and ND [54,55], and their contribution in AD pathogenesis needs further investigation. The diverse and obscure points of neuroinflammation and ND [56] could also be clarified. Furthermore, clinical trials on AD and other ND have failed to provide hopeful results [57] for diverse causes, including the relevant role of sex/gender dimorphism (which also justifies the differences observed in the onset, progression, and hallmarks of neuroinflammation in the various NDs), which will be described in the following section [58].

### 2.3. Considerations on Immune Cells Infiltrating the CNS and New Evidence on the Migration of Immune Cells outside the CNS

The mechanisms involved in the infiltration of peripheral immune cells into the CNS during neuroinflammation and ND have been gaining great interest in recent years, leading to the development of numerous therapies able to modulate immune cells at the BBB, the choroid plexus (ChP) epithelium, and glial barrier. For instance, natalizumab therapy, a drug inhibiting the adhesion and trafficking of monocytes and clonotypic cells across the BBB, has been used for almost two decades to treat MS [59,60].

Furthermore, fundamental CNS immune cell populations, i.e., dendritic cells (DCs), T cells, B cells, and other myeloid cell populations, have been found to migrate out of the CNS and mediate signals from the CNS to peripheral lymphatics [61]. This has been supported by recent evidence reporting the involvement of the meningeal lymphatic system not only in fluid homeostatic CNS functions but also in allowing immune cell migration and facilitating DC migration from the CNS to the meningeal borders and draining cervical lymph nodes [61].

However, work needs to explicate the function of each CNS-associated lymphatic region in overall CNS immunity. The results obtained would accelerate the development of new therapies to modulate the interplay between lymphocytes and leukocytes and consequently treat cases with CNS diseases.

### 2.4. Sex/Gender Dimorphism: An Important Modifier of Immune System and Physiology of Brain, and a Crucial Differential Driver in Diseases

The term “sex” indicates the diverse biological and physiological features of male and female individuals, the term “gender” refers to the social and cultural differences between men and women [58]. Biological, socioeconomic, and cultural differences impact the health and diseases of individuals. Dissimilarities in the anatomy and physiology of the body systems characterize women and men, while gender influences the norms that socially impose and specify roles and relationships among the individuals of a precise society and time. Hence, biological distinctions in the morphology and functions of the nervous system exist between the sexes, as proven by studies in human and animal models [58]. Specifically, amygdalae are larger in males than in females. While the dimorphism controls emotional memories in the female amygdala and implicates the involvement of the left region (visually predominant, positive, and negative emotions), in males, they activate the right region of the brain (negative emotional responses). Furthermore, prefrontal cortical regions have higher levels of estrogen receptors. Such could clarify the diversity in decision-making between the two sexes. Structural neuroimaging investigations have also confirmed the presence of reduced grades of overall cortical thickness and increased cortical thickness decline in men and greater white-matter volume in women. Moreover, differences in neurotransmitter systems (i.e., adrenergic, serotonergic, cholinergic systems, and corticosterone, benzodiazepine, and cholecystokinin, factors largely associated with episodic memory) characterize the two sexes. Higher levels of serotonin are more typical of men than women and may impact disease conditions related to serotonin dysfunction [58]. These diversities can in turn determine variations in the learning process, as identified during stress conditions, as well as subsequent habituation (increased in males but restrained in females). Such variation has been associated with the diverse levels and profiles of sex hormones in the two sexes. Accordingly, interesting results have been found in studies on cognitive decline and neurodegenerative and psychiatric diseases conducted in human and animal models. They report that sex hormones alter the permeability of the BBB, which represents one of the key pathophysiological ND hallmarks. Such findings could illuminate these disease processes; however, further research is required for proving and supporting this relationship [58].

Sexual dimorphism also influences the immune system of both sexes, with typical and excessive responses of both innate and adaptive immunity versus pathogens and endogenous antigens in females than males. This impacts the outcomes of infections and the efficiency of vaccines [62], and simultaneously disposes females to a higher risk of autoimmune diseases, even if the mechanisms related to these modifications are not yet fully explained. Furthermore, the close relationship between variations in number and functions of immune cells, as well as in the levels of cytokines or other systemic immune mediators, and the biological consequence of sex chromosomes and sex hormones is well recognized [58]. Therefore, sex designates an important trigger of the physiological and pathological conditions of an organism, humans included, which acts via genetic, epigenetic, and hormonal regulations. This is object of study of sex/gender medicine, born in the late 1990s, to identify fluctuations between diseases and their determinants in the two sexes [58]. Variations in the mechanisms and pathways related to the pathophysiology of diseases have been observed between the two sexes, as well as in their clinical manifestation, prognosis, and outcomes. Accordingly, ongoing studies on such aspects of major diseases are being performed [58,63]. They can prove useful in establishing new criteria and guidelines for the two sexes, particularly women. Women are more challenging to diagnose, and the traditional diagnostic tests, created for men, have a lower sensitivity and specificity in quantifying the biomarkers in female blood samples. Thus, new protocols are imperative. However, clinicians and researchers have until now paid little attention to sex and gender in health planning and medical practice.

Therefore, numerous efforts are required to incorporate sex and gender in modern medical research, clinical trials, clinical practice, and medical societies and institutions. Finally, different gender variables need to be studied at all levels and by biomedical and pharmaceutical organizations for the development of distinctive biomarker panels and correct therapies for both sexes.

## 3. Changes in the MGB Axis and Neuroinflammation

Gut microbiota and the CNS are influenced by the MGB axis (see Figure 1), discovered in 2012 [64], and constituted by neuroanatomical brain structures and intestinal nerves, i.e., the vagus nerve, located in the intestinal wall [64]. The vagus nerve mediates a response of the descending branch, which in turn controls intestinal activities. In addition, the hypothalamic–pituitary–adrenal (HPA) axis represents another component of the MGB axis [65]. The HPA axis monitors the changes in the composition and functions of the gut microbiome. Thus, HPA dysfunctions result in MGB alterations related to the pathogenesis of neuropsychiatric diseases. Specifically, HPA activation results in the induction of inflammatory signaling pathways, releasing inflammatory mediators, such as tumor necrosis factor α (TNF-α), interferon γ (IFN-γ) and interleukin 6 (IL-6) [66]. In turn, these mediators contribute to damaging BBB integrity and the onset of brain diseases via systemic circulation and simultaneously to alter the gut mucosal barrier. Moreover, the inflammatory response induced via the HPA axis impacts the secretion of glucocorticoids [67], modulating gut function and production of proinflammatory factors [68]. This vicious cycle also evokes the activation of enteric immune cells, such as Th17 and NK cells, which infiltrate the brain, causing neuroinflammation [69]. Neuroinflammation, in turn, additionally contributes to modifying the gut microbial composition, and this further provokes activation of enteric immune cells and release of microbiota-derived metabolites, i.e., lipopolysaccharide (LPS). These exacerbate the bidirectional via of inflammatory signals, contributing to the onset of dysbiosis, a typical alteration in gut microbiota and the MGB axis. Dysbiosis has recently attracted increasing interest for its pathogenic role in immune-mediated diseases, including metabolic syndrome, gastrointestinal tract infections and inflammatory bowel disease, as well as autoimmune diseases such as systemic lupus erythematosus, systemic sclerosis, Sjogren’s syndrome, antiphospholipid antibody syndrome, multiple sclerosis (MS) and myasthenia gravis (MG). MG is a typical neuromuscular autoimmune disease triggered by immune-mediated damage to the neuromuscular junction (NMJ), and with pathogenesis likely multifactorial [70]. Perturbations in human microbiota have been described to be related to MG pathogenesis and clinical course. MG cases compared with age-matched controls show a characteristic composition of the oral and gut microbiota, with a typical increase in *Streptococcus* and *Bacteroides* and a reduction in *Clostridia*, as well as a reduction in short-chain fatty acids. Moreover, it has been demonstrated that restoration of gut microbiota disorder after probiotic administration determines an improvement in symptoms in MG cases [7].

Such evidence highlights the double role of the MGB axis in maintaining host health and contributing to the typical alterations causing dysbiosis and MGB axis disorder, and to the onset of MG and some NDs, such as PD and AD. However, the related molecular and cellular mechanisms are not clear. Some studies also evidence that behavioral phenotypes can be transmitted from humans to animals via transplantation/translocation of the gut microbiota [7]. This emphasizes the role of MBG alterations in ND. However, further research is needed to confirm if the discoveries in animals may be also obtained in humans to identify all the relevant mechanisms by which the gut microbiota controls neuroinflammation and ND. Such studies could allow the development of new microbiota-based strategies for diagnosis, treatment, and clinical management of neuroinflammation and ND.

## 4. Autophagy

The term “autophagy” indicates a cell death process, which is physiologically regulated and evolutionarily conserved. It causes degradation of cytoplasmic proteins and other macromolecules within the lysosome in multicellular organisms [71]. It has been demonstrated to be involved in neuroinflammation and ND, which are characterized by protein aggregation and exacerbated autophagy [72,73,74]. However, the mechanisms involved are not clear [75,76,77]. Despite this, some drugs, including cocaine [78] or other toxic substances of exogenous or endogenous nature and pathogens have been tested to evoke autophagic cell death in astrocytes and in consequent pathogenesis of neurodegeneration [79]. Recently, a close relationship between glia maturation factor (GMF), autophagy-related proteins, and the NLRP3 inflammasome and a shift of microglia from M1 to M2 in AD patients has been detected [80,81]. However, further investigations are needed for understanding the role of autophagy in ND or neuroinflammation-associated disorders.

## 5. Ferroptosis in Neuroinflammation

Ferroptosis, discovered in 2012 by Brent R. Stockwell, is a new form of regulated cell death (RCD) described by the accumulation of lethal amounts of iron- and lipid-dependent reactive oxygen species [82]. Ferroptosis results in different morphological and biochemical characteristics from other conventional RCD forms [82]. It is evoked by severe peroxidation of membranes containing polyunsaturated fatty acids (PUFAs), and regulated by lipid, iron, and amino acid metabolism and signaling transduction. The critical phases involved in ferroptosis include the accumulation of intracellular free iron, glutathione depletion, and peroxidation of PUFA-rich membranes [82,83]. Recent evidence has documented ferroptosis as a crucial factor in the pathogenesis of several diseases, such as ND [82,83,84,85]. Accordingly, iron homeostasis, oxidative stress, and subsequent neuroinflammation have been described to contribute to the regulation of ferroptosis and neuronal health [82,83,84,85]. However, the precise molecular mechanisms underlying the involvement of ferroptosis in the pathological processes of neurodegeneration and its impact on neuronal dysfunction remain to be discovered. Nevertheless, ferroptosis has recently been reported to likely be regulated by ELA/APJ signaling of the apelinergic system [13], where ELA is a peptide hormone belonging to the adipokine group and a component of the apelinergic system, discovered in 2013–2014 [13]. This relationship, mediated by ELA/APJ signaling, might be a promising strategy for the treatment of NDs, such as stroke [13]. Accordingly, a recent study in mouse models of middle cerebral artery occlusion (MCAO) has demonstrated the protective role of the ELA–APJ axis in ischemic stroke after treatment with ELA-32 (widely quoted in [13]). A reduction in cerebral ischemic lesion and an improvement in neurobehavioral and cognitive deficits have been detected. Furthermore, ELA-32 administration has been revealed to ameliorate neuronal ferroptosis, iron deposition, mitochondrial damage, lipid peroxidation, and glutathione reduction. These results have emphasized the role of the ELA–APJ axis in attenuating neuronal ferroptosis after ischemic stroke (widely quoted in [13]). However, further data are needed to provide other/novel strategies to modulate the onset of neuroinflammation and ND, such as stroke.

## 6. The Close Link of Endothelial Dysfunction with Neuroinflammation and ND

Endothelial dysfunction represents another condition contributing to neuroinflammation and ND, which occurs with typical cellular and molecular mechanisms, including changes in the glycocalyx [86,87]. Such a close relationship of damaged endothelium with neuroinflammation is related to the relevance of the endothelium in the brain; it is a fundamental component of the neurovascular unit (NVU), composed of ECs arranged with neurons, glial cells, and other vascular elements [86,87]. NUV mediates diverse functions: maintenance of CNS homeostasis, physiological neurotransmission, and neuronal survival [62]. Furthermore, EC and glial cells, such as microglia cells, contribute to BBB integrity and provide both nutrients and oxygen. In systemic infections or in the presence of systemic inflammation, circulating toxins and inflammatory mediators infiltrate ECs and consequently the BBB. Accordingly, any alteration or disorder of the NVU also involves the BBB and causes neuroinflammation, which in turn contributes to evoking age-associated cognitive deficits and consequently ND onset [86,87,88]. Frequently, the altered clearance of amyloid-β peptide and its consequent accumulation in the brain constitute the typical trigger of NVU. In this case, the release of toxic small molecules and inflammatory products that cross the damaged BBB determines neuroinflammation [62]. However, cardiovascular disorders, including cerebrovascular diseases, i.e., macro-infarcts, lacuna, microbleeds, atherosclerosis, arteriolosclerosis, and cerebral amyloid angiopathy (CAA), have been documented to directly contribute to NVU dysfunction [62,86]. Among these, microvascular diseases have been demonstrated to affect NVU by determining alterations in the physiological process of brain oxygenation, as well as reduced blood flow and subsequent hypoxia [62,86,87,88]. Accordingly, chronic hypoxia–ischemia is accepted as a key trigger of chronic NVU damage and BBB dysfunction related to many NDs, such as stroke, MS, AD, and PD; however, there are increasing data linking BBB breakdown to physiological aging processes, specifically with vascular aging. This initially involves the hippocampus in subjects without cognitive impairment, and occurs more rapidity in old people and remarkably with concomitant MCI [86,87,88]. During normal aging, BBB dysfunction affects the CA1 region and the dentate gyrus, but not the CA3 region [86,87,88]. Moreover, hippocampal BBB distribution has been found to precede the onset of hippocampal atrophy [86,87,88]. Analysis of cerebrospinal fluids from MCI cases when compared with cognitively normal persons has evidenced a significant increase in pericyte damage biomarkers (i.e., platelet-derived growth factor receptor (PDGFR)-β) by implying an immediate role of pericytes, as opposed to other cell types, in BBB breakdown [86,87,88]. The involvement of BBB dysfunction in AD has been related to a reduced presence of tight junctions and an abnormal morphology in brain ECs. In addition, an altered diameter of blood vessels after typical tau deposition has been detected [86,87,88]. An anomalous angiogenesis in AD cases, likely due to altered function and quantity of trophic factors, has also been assessed. Patients with AD have expanded levels of VEGF, both in serum and temporal cortex and hypothalamus, and decreased expression of both VEGFR-1 and VEGFR-2 [86,87,88]. Some studies have pointed to VEGF itself being the cause of such decreases in levels of the two receptors. Indeed, VEGF mediates such effects through a ligand-mediated endocytosis mechanism [86,87,88]. Thus, VEGF in AD has a role of antagonist versus its receptors, resulting in an altered angiogenesis. Moreover, in vitro Aβ accumulation has been demonstrated to be able to reduce the mRNA levels of VEGFR-1 and VEGFR-2, resulting in increased VEGF reactivity [86,87,88].

Likewise, ALS patients show alterations in NVU and angiogenic factors, including VEGF. This finds confirmation in studies conducted in both SOD-1-mutant mice and ALS patients. Reduced levels of tight junctions, such as ZO-1, and BBB breakdown, which precede motor neuron death, have been detected in such studies. This suggests a key role of vascular damage as an early pathogenic ALS mechanism [62,86,87,88,89], data also validated by high levels of metalloproteinase (MMP)-2 and MMP-9 in peripheral blood samples from ALS cases and by the results obtained by Nicaise and colleagues on variations in the composition of vascular NVU elements in the early ALS stage [62]. The SOD-1 mouse model has also evidenced a blood–spinal cord barrier (BSCB) dysfunction, characterized by ex-erythrocyte extravasation, neurotoxic hemoglobin accumulation, and NUV injury via iron-dependent oxidative stress [62]. Studies in G93A SOD1 mice have demonstrated alterations in the NVU that are not only structural but also functional, as confirmed by a downregulation of Glut-1 and CD146 expression early and late in the disease [62]. Compared to healthy controls, ALS patients have also been demonstrated to have elevated levels of VEGF, particularly VEGF-A, in the blood and CSF [62], possibly due to a compensatory mechanism. Investigations into SOD-1-mutant mice have also revealed that VEGF-A also exercises neuroprotective effects by decreasing MN cell death via activation of the PI3K-Akt pathway [62], and this may result in a delay of disease onset [62,86,87,88,89]. However, other studies have proven the presence of reduced levels of VEGF and its receptor VEGFR in ALS cases and in subjects homozygous for certain haplotypes, i.e., three polymorphisms in their genes (−2578 C/A, −1154 G/A, and −634 G/C) [62]. The reason has been attributed to the destabilization of VEGF mRNA induced by SOD1 protein [62].

This evidence globally suggests that the maintenance of BBB and NVU integrity, as of entire cardiovascular system, could ameliorate the health of the cerebrovascular system and represent the best avenue for the development of potential strategies for improving blood flow at the cerebral microvascular level by protecting the BBB and NVU. Preserving the integrity, permeability, and function of the BBB and NVU could stop or delay the progression of neuroinflammation and ND. To achieve this goal, it is imperative to identify all the pathways involved in the pathophysiology of these diseases, and particularly those related to BBB and NVU dysfunction. Surely, this objective can be realized by performing multiple omics investigations, offering the opportunity of acquiring major, relevant, and new data. Accordingly, such studies are encouraged.

## 7. miRNAs and Epigenetic Factors in Neuroinflammation and ND

Recently, the modulation of genes related to neuroinflammation has been considered as a means to mitigate it. MicroRNAs and other epigenetic factors, universal regulators of differentiation, activation, and polarization of all the cells of human body, including immune and neuronal cells, appear to be directly responsible for neuroinflammatory processes. Recent investigations demonstrate different expression levels of miRNA and epigenetic factors in microglia, both in normal and inflamed CNSs, suggesting their role in brain health and neuroinflammation-associated disorders [90]. Cases of epilepsy and neuroinflammation in the hippocamp of patients with sclerosis have shown low levels of mature micro-RNAs in human temporal lobes [91,92]. Among these, microRNA-155 appears to negatively regulate BBB function in chronic neuroinflammation and neurodegeneration [93]. miR-195 inhibits autophagy after peripheral nerve damage [94]. In contrast, microRNA-188-3p, in an upregulated state, constrains the neuroinflammation and recovers memory in AD patients [95]. miR-137 attenuates beta-induced neurotoxicity in Neuro2a cells [96]. miR-124 expression modifies promoter DNA methylation and microglial functions [97]. Notably, microRNA-30e controlled neuroinflammation via NLRP3 in an MPTP-induced PD model [98]. MicroRNA-129-5p exacerbates neuroinflammation and BBB injury [99]. Similarly, miR-17-92 triggers the differentiation of neurons during neuroinflammatory conditions [100]. MicroRNA-139 favors AD pathogenesis via cannabinoid receptors [101]. Thus, microRNAs constitute good therapeutic targets to produce novel anti-neuroinflammatory AD treatments [90].

Moreover, other epigenetic factors related to post-transcriptional RNA changes modulate mRNA coding properties, stability, and translatability, expanding the genome’s coding capacity. They appear to influence neuroinflammation. Among these, A-to-I RNA editing, m6A RNA methylation, and alternative splicing (AS) impact the neuronal cell life cycle, induce neuron death mechanisms, and contribute significantly to neuroinflammation and age-related neurodegeneration [17]. A-to-I RNA editing is a post-transcriptional mechanism modulating double-stranded (ds) RNA structures via the catalytic activity of adenosine-deaminase acting on RNA (ADAR) enzymes. It consists in the deamination of specific adenosine (A) into inosine (I) by altering both coding and non-coding transcripts [17]. Three ADAR enzymes are expressed in human cells–ADAR1, ADAR2 and ADAR3–and have high expression and activity in the brain in terms of regulating neurodevelopment, brain function, and physiological brain aging (widely quoted in [17]). Consequently, the brain appears to be susceptible to ADAR activity and RNA-editing dysregulation, which potentially initiate CNS disorders, such as glioblastoma, epilepsy, and ND (widely quoted in [17]).

Altered expression of N6-methyladenosine (m6A), a dynamic and reversible post-transcriptional alteration adding a methyl group to the N6 position in selected adenosines of each type of RNA [17], has been documented in aging mouse and human brains. In terms of ND, unusual m6A alterations have been identified in AD, PD, and ALS.

ND patients, including mainly AD, PD, ALS, frontotemporal dementia (FTD) and familial dysautonomia (FD) cases, have AS alterations [17].

The close relationship of post-transcriptional RNA modifications with brain aging and neurodegeneration emphasizes the possibility to reduce or inhibit these processes; antisense oligonucleotides (ASOs) can modify their expression. ASOs appear to eliminate causative splicing defects in PD, AD, FTD and ALS (widely quoted in [17]).

The growing evidence on the contribution and serious impact of A-to-I RNA editing, m6A RNA methylation, and alternative splicing on brain aging process, neuroinflammation, and ND points to the need for further investigations on these processes and how they may impact each other so as to control them simultaneously.

## 8. Transcriptional Factors and Related Pathways: Focus on NF-kB (Nuclear Factor Kappa-Light-Chain Enhancer of Activated B Cells) and Related Pathways

Other modulating factors of neuroinflammation are transcriptional factors, able to activate an inflammatory network in the brain and all linked to the NF-κB pathway, an ancient signaling pathway specialized in host defense [102]. The NF-κB pathway is a cytoplasmic molecular complex of diverse proteins comprising the Rel family proteins RelA/p65, c-Rel and RelB and NF-κB components-p50/p105 and p52/p100, and is commonly inhibited by binding to IκB proteins (i.e., IκBα, IκBβ, IκBγ, IκBδ, IκBϵ, IκBζ and Bcl3) via the action of many signaling pathways and negative feedback loops regulating diverse mechanisms at various levels of the signaling cascades. Immune insults and external and internal danger signals, such as oxidative and genotoxic stress and tissue injury, constitute its activators [102]. In addition, Toll-like receptors (TLRs) and inflammasome [103,104,105], as well as several upstream kinase cascades via canonical or non-canonical pathways, can activate the NF-κB pathway. IKKα/β and NIK are the most important upstream kinases. IKKγ is generally referred to as a nuclear factor-kappa B essential modulator (NEMO), an important regulatory component of the IKK complex linked upstream to genotoxic signals and IL-1 and TNF receptor-mediated signaling [103,104,105]. NF-κB complex activation, playing the crucial role of a pleiotropic mediator of gene expression, determines its translocation into the nucleus and the expression of target genes, encoding various molecules, such as proinflammatory cytokines, chemokines, adhesion molecules, eicosanoids, growth factors, metalloproteinases, nitric oxide, etc. [102]. NF-κB signaling has been reported to be one of the major pathways stimulating neuroinflammation [102,106].

Recent studies have evidenced the beneficial effects of dietary supplementation with anti-inflammatory compounds on cognitive decline, neuroinflammation and oxidative stress by acting on the NF-kB pathway in AD-like animal models [107,108]. Curcumin, krill oil, chicoric acid, plasmalogens, lycopene, tryptophan-related dipeptides, hesperidin, and selenium peptides have been tested, despite their heterogeneity, and have shown helpful actions on cognitive deficits and LPS-induced neuroinflammatory responses in rodents by affecting the NF-κB pathway [106,107,108]. Overall, dietary interventions could represent positive factors in countering AD, or other ND, by acting on neuroprotection and immune regulation. For example, treatment with metformin, an antidiabetic drug, has demonstrated anti-inflammatory effects via many mechanisms, revealing its potential as a therapy for neuroinflammation.

However, as evidenced in such reviews, the mechanisms involved in neuroinflammation are various and complex: numerous molecules are combined in a network and consequently can modify each other. For example, metformin significantly prevents nuclear translocation of p65, but pretreatment with compound C, an AMPK inhibitor, eliminates this effect, while silencing HMGB1 abolishes NF-κB activation. SIRT1 deacetylates FoxO, increasing its transcriptional activity. mTOR in dendritic cells regulates FoxO1 through AKT. Interactions between the various molecules need to be further explored to clarify their specific mechanisms and provide more guidance for the treatment of neuroinflammation [109].

Based on the evidence described above, mTOR and AKT pathways, as well as JAK-STAT, and PPARγ, and Notch pathways, constitute other crucial pathways in neuroinflammation [9,110,111,112,113,114,115]. They represent highly conserved signaling hubs that coordinate neuronal activity and brain development and participate in neuroinflammation. Accordingly, hyperactivation of JAK/STAT and mTOR and inhibition of PPARγ and AKT signaling have been associated with various neurological complications, including neuroinflammation, apoptosis, and oxidative stress [112,113,114,115]. Remarkably, target modulators have also been described to act during acute and chronic neurological deficits. For example, natural products, such as osthole, an important ingredient of traditional Chinese medicinal plants often found in various plants of the Apiaceae family, have been shown to target these pathways [116]. Osthole induces neurogenesis and neuronal function via the stimulation of Notch, BDNF/Trk, and P13k/Akt signaling pathways. This upregulates the expression of various proteins, such as BDNF, TrkB, CREB, Nrf-2, P13k, and Akt, and inhibits MAPK/NF-κB-mediated transcription of genes involved in the production of inflammatory cytokines and the NLRP-3 inflammasome. Thus, modulation of Notch, BDNF/Trk, MAPK/NF-κB, and P13k/Akt signaling pathways by osthole confers protection against neuroinflammation and ND [116].

Te evidence described above suggests the neuroprotective potential of several compounds and natural products as possible therapeutic agents for neuroinflammation and NDs. However, a limitation of some of these substances is their low bioavailability and solubility in water. Furthermore, the use of innovative nanotechnology or the incorporation of a more polar group would be advantageous to increase the bioactivity and physicochemical properties of such compounds or natural products, such as osthole. To this end, liposomes, microspheres, nanoparticles, transferosomes, ectosomes, lipid-based systems, etc. have been developed for the modified delivery of various herbal drugs. For example, osthole-loaded nanoemulsion has been reported to effectively target the brain and have beneficial effects in the treatment of AD. Therefore, the development of potential nanocarriers such as liposomes, microspheres, and nanoemulsions could improve the bioavailability of such compounds [107,108]. However, further studies are needed to evaluate the real therapeutic effect of such compounds on neuroinflammation.

## 9. Circadian Cycle and Neuroinflammation

Another determinant of neuroinflammation is alteration in circadian cycle/rhythm, a fundamental process of life developed during the long-term evolution of organisms. It has diverse functions, maintaining the proliferation, migration, and activation of cells, and particularly of immune cells [117,118]. Circadian rhythm disorders impact the onset and development of neuroinflammation by activation of glial cells and peripheral immune responses [18,118,119]. Animal models exposed to nightshifts or night light have been confirmed to have significant levels of activated microglia and proinflammatory cytokines in brain [120,121]. Sleep deprivation has also been demonstrated to trigger the transcriptional factor NF-κB and intensify the release of IL-1β and TNF-α in the hippocampus, resulting in neuronal injury [122]. Studies have also revealed high mRNA levels of IL-1β and TNF-α in brain tissue of experimental animals, which evoke significant alterations in circadian rhythm, responsible for modifications in the sleep–wake cycle [123]. Inhibition of such cytokines has resulted to reduce spontaneous non-rapid eye movement sleep in experimental animals [124,125], confirming that proinflammatory cytokines induce effects on the circadian cycle and neuroimmune function. In addition, circadian system disorders influence microglial activation and their phenotypes [120,121,122]. Accordingly, under conditions of light exposure, diverse investigations in rats report an increased inflammatory activity of microglia, accompanied by significant rises of TNF-α, IL-1β, and IL-6 [120,121,122,126].

A critical role of circadian cycle/rhythm has been evidenced in the regulation of the peripheral immune system [127], including innate and adaptive cells. They have their own molecular clock and display significant rhythmic differences during recruitment and activation processes [128]. A regulation of the bone marrow chemokine CXCL12 on the hypothalamic sympathetic–parasympathetic nervous system in a circadian manner has also been detected. It determines periodic fluctuations in CXCL12 levels and CXCR4 receptor activation to sustain daily rhythmic changes in the number of neutrophil cells in the bone marrow blood reserve [129,130]. In macrophages, the circadian cycle impacts their pattern-recognition receptor signaling pathways, inflammatory mediators, and phagocytic activity [131]. Krüppel-like factor 4 (KLF4), whose expression is time-regulated, appears to regulate the macrophage phenotype and rhythmic expression of inflammatory factors [132]. In addition, the REV-ERBα clock gene has been demonstrated to modulate the expression of the PI3K/Akt signaling pathway and the regulation of the diurnal rhythm of macrophage polarization [133]. Thus, REV-ERBα also represents a potential target for regulating circadian rhythms and inflammatory response. Similarly, adaptive immune cells also display rhythmicity, with immune responses that differ significantly during the different hours of the day. For example, more CD8+ T cells are produced during the day than at night in response to antigen immunization, and the rhythmic response dissolves by knocking out the Bmal1 gene in T cells, further validating the relevance of circadian rhythms in modulating adaptive immune responses [134].

## 10. Chronic Low-Grade Inflammation and Neurodegenerative Diseases

Currently, the precise nature and temporal characteristics of the relationship between neuroinflammation and ND remain largely unknown. Clinical and preclinical studies have described how systemic chronic inflammation (SCI) should be considered a potential driver of the onset of the neurodegenerative process associated with cognitive impairment [135,136]. Several studies have proposed the concept of chronic low-grade inflammation as potentially causal in the etiopathogenesis of dementia and other ARDs of the elderly individual, and the term “inflammaging” has been coined for this phenomenon [137,138]. Specifically, inflammaging refers to the presence of chronic low-grade systemic inflammation that occurs during aging in the absence of overt infection (the so-called sterile inflammation). Clinical and epidemiological studies have shown that this process is a relevant risk factor for morbidity and mortality in the elderly [137,138]. In particular, the presence of SCI leads to an increased risk of metabolic diseases (e.g., hypertension, diabetes, dyslipidemia) and osteoporosis, cancer, and cardiovascular, neurodegenerative, and autoimmune diseases [135].

SCI implies the involvement of several cytokines and transcription factors that regulate chronic inflammation at the tissue and causal levels for different ARDs. Among the cytokines, IL-6 is probably the one most associated with a robust chronic inflammatory response that characterizes different ARDs [139]; other inflammatory cytokines that participate in the inflammatory process during ARD are IL-1β and TNF-α [139,140]. In turn, cytokines interact with specific tissue surface receptors, regulating the inflammatory cascade by regulating transcriptional processes. The two main protein transcription factors associated with SCI are STAT (signal transducer and activator of transcription) and NF-κB [102]. These proteins regulate a series of genes that code for the formation of inflammatory cytokines.

Over the last decade, the role of low-grade SCI in periodontal disease (PeD) has been suggested as a potential risk factor for overall dementia and particularly AD. Several authors have described the presence of significantly elevated antibody levels toward specific oral cavity opportunistic pathogens causing PeD in subjects with AD but also MCI compared with control subjects without cognitive impairment [141]. Regarding specific oral pathogens, the one most implicated in the link between dementia and PeD appears to be porphyromonas gingivalis [142], but significantly elevated levels of oral microbial load of other pathogens such as fusobacterium nucleatum and treponema denticola have been described in subjects with AD and MCI compared with control subjects [142]. Data from a recent national US retrospective cohort study showed that periodontal pathogens increase the risk of AD incidence and mortality [143]. In addition, data from a recent meta-analysis showed that the risk of cognitive disorder in individuals with PeD increases as the severity of PeD increases, and this risk appears to be greater in the female sex [144]. There are at least two main mechanisms by which PeD can cause cognitive disorders. The first involves the presence of an increased cerebral inflammatory state caused by the SCI process originating from oral pathogens; the second involves a direct action of periodontal bacteria on the CNS that cross the BBB and cause its breakdown with subsequent, potential triggering of the preexisting neurodegenerative process [141,142,143,144].

In addition to increased risk of dementia, some studies have suggested that PeD may increase the risk of PD [145]; however, data from a recent meta-analysis revealed no association between PeD and increased risk of PD [145]. In conclusion, PeD is associated with an increased risk of overall dementia, AD, and MCI, and this appears to be due to low-grade SCI sustained by the oral pathogens that cause PeD. However, prospective data on large population cohorts are needed to confirm the role of PeD as a risk factor for AD, dementia, and possibly other neurodegenerative diseases. If confirmed, such data will have major implications for the treatment and prevention of cognitive disorders.

## 11. Conclusions

**Figure 2:**
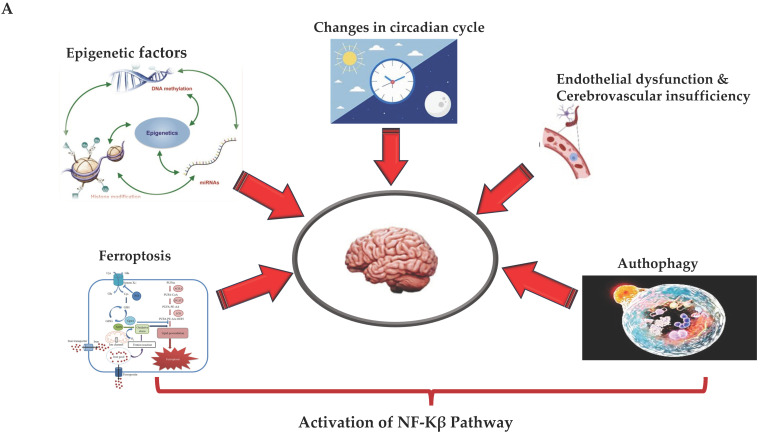

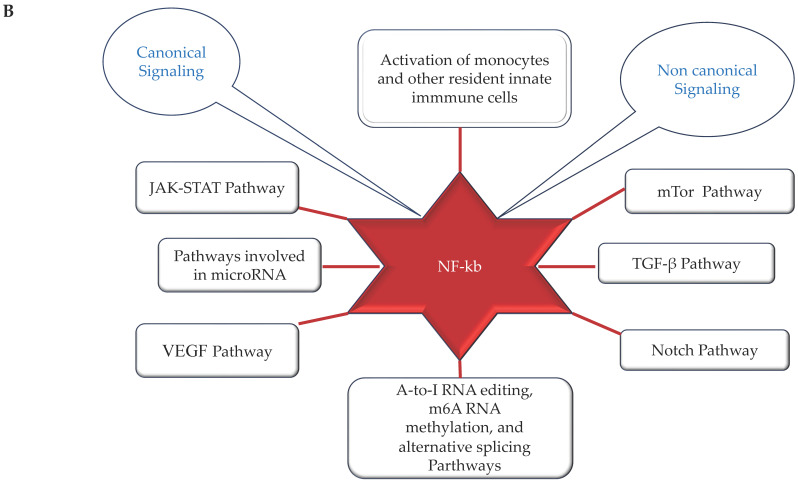
(**A**,**B**) (by Biorender software): Model describing the novel mechanisms involved in neuroinflammation and its relation to the onset of ND. In (**A**), it illustrates how the ferroptosis, autophagy, epigenetic factors, changes in circadian rhythm, and endothelial dysfunction associated with cerebrovascular insufficiency determine all the activation of NF-kB pathway through canonical or non-canonical signaling. In (**B**), it shows how activation of the NF-kB pathway through canonical or non-canonical signaling can activate a network of different signaling pathways, all related to the onset of neuroinflammation and the consequent onset of ND.

With this review, we have provided an overview of the new mechanisms associated with the relationship between neuroinflammation and subsequent onset of ND (see Figure 2A,B). The latter offers the possibility of hypothesizing and developing new treatments and identifying diagnostic and prognostic biomarker profiles for neuroinflammation and ND. They could include the assessment of transmigration and activation levels of monocytes, as well as the levels, activation, and quantification of clonotypic cells and their mediators, and the evaluation of expression of NF-kB and other transcriptional factors (i.e., ERG factor) and the related pathways [146,147]. The concomitant assessment of microRNAs and epigenetic factors involved in the regulation of these mechanisms could be additionally helpful. Furthermore, the targeting of autophagy and ferroptosis is gaining more and more interest, as it contributes to the modulation of neuroinflammation and the onset of ND, as well as to endothelium-related BBB dysfunction. Considering all the findings to date, the complex pathophysiology and pathogenesis of neuroinflammation and ND might appear clearer, as well as all pathways and cells. This could facilitate the identification of biomarkers and targets and, consequently, the management of NDs. Therefore, further efforts and investigations are needed. Advances in omics methodologies, artificial intelligence, machine learning, advanced biological techniques, metagenomics, and meta-transcriptomics are currently important in neuroscientific research and could be used to achieve this goal. Similarly, large-scale randomized controlled trials are needed. Such studies would pave the way for next-generation treatment strategies capable of modulating neuroinflammation during ND.
